# Lower gray matter volumes of frontal lobes and insula in adolescents with anorexia nervosa restricting type: Findings from a Brain Morphometry Study

**DOI:** 10.1192/j.eurpsy.2020.19

**Published:** 2020-03-16

**Authors:** O. Curzio, S. Calderoni, S. Maestro, G. Rossi, C. F. De Pasquale, V. Belmonti, F. Apicella, F. Muratori, A. Retico

**Affiliations:** 1 Institute of Clinical Physiology of the National Research Council (IFC-CNR), Department of Biomedical Sciences, Pisa, Italy; 2 Department of Clinical and Experimental Medicine, University of Pisa, Pisa, Italy; 3 Department of Developmental Neuroscience – IRCCS Fondazione Stella Maris, Pisa, Italy; 4 G. Monasterio Foundation, Tuscany Region (FTGM), Pisa, Italy; 5 Pisa Division, INFN - National Institute for Nuclear Physics, Pisa, Italy

**Keywords:** adolescence, anorexia nervosa restricting type, brain structures, region of interest analysis

## Abstract

**Background.:**

Brain atrophy in anorexia nervosa (AN) is one of the most marked structural brain changes observed in mental disorders. In this study, we propose a whole brain analysis approach to characterize global and regional cerebral volumes in adolescents with restricting-type anorexia nervosa (AN-r).

**Methods.:**

A total of 48 adolescent females (age range 13–18 years) were enrolled in the study (24 right-handed AN-r in the early stages of the illness and treated in the same clinical setting and 24 age-matched healthy controls [HC]). High-resolution T1-weighted magnetic resonance images were acquired. Cerebral volumes, including the total amounts of gray matter (GM), white matter (WM), and cerebrospinal fluid (CSF) were obtained with the Statistical Parametric Mapping software (SPM8); specific cortical regional volumes were computed by applying an atlas-based cortical parcellation to the SPM8 GM segments. Analysis of variance (ANOVA) was performed to identify any significant between-group differences in global and regional brain volumes.

**Results.:**

The analyses revealed reduced total GM volumes (*p* = 0.02) and increased CSF (*p* = 0.05) in AN-r, compared with HC. No significant between-group difference was found in WM volumes. At the regional level, significantly lower GM volumes in both frontal lobes (*p* = 0.006) and in the left insula (*p* = 0.016) were detected. No significant relationships were found between cerebral volumes and duration of illness, psychiatric comorbidities, psychopharmacological treatment, prepubertal phase, or presence of amenorrhea.

**Conclusions.:**

The topographic distribution of GM reduction in a homogenous group of AN-r involves regions responsible for the emotional and cognitive deficits associated with the illness. These findings are discussed in relation to the roles of the insular cortex and the frontal lobes.

## Introduction

Acute reduction of brain tissue in anorexia nervosa (AN) is one of the most evident structural brain changes that can be observed in mental disorders. Most studies have reported reduced brain volume and increased cerebrospinal fluid (CSF) in AN as an effect of starvation on the brain [1–7]. Brain magnetic resonance imaging (MRI) in patients with AN consistently showed sulcal widening and ventricular enlargement [8] correlated with the reduction of total gray matter (GM) and white matter (WM) volumes. The interaction between low weight, duration of illness, and brain changes remains controversial [8–12]. However, most of these studies have focused on adults. The onset of eating disorders frequently occurs during adolescence, and studies on adolescents with AN are typically less biased by illness duration [12–19]. A meta-analysis by Seitz and colleagues [4] on recovered patients with AN systematically examined potential residuals of volumetric brain alterations. Different time courses were described for GM and WM recovery, pointing to the possible involvement of different underlying mechanisms. GM changes seemed to be more pronounced in adolescent patients, while WM changes did not show any age-related effect. Seitz et al. [4] suggested that greater GM plasticity in the developing adolescent brain could lead to greater susceptibility to starvation effects. The same meta-analysis concluded that in adolescent anorectic patients, GM is more affected than WM [4]. It is a matter of debate whether brain abnormalities in AN are secondary to starvation or indicative of a primary disturbance preceding the illness and representing an underlying biological substrate [20,21]. Moreover, there are conflicting results regarding the reversibility [17,22] or the persistence of brain abnormalities when weight is restored [9,23]. An earlier study using SPECT showed that individuals with teenage-onset AN, most of whom were weight-restored, display marked hypoperfusion of temporal, parietal, occipital, and orbitofrontal lobes compared with the control group, and that regional cerebral blood flow was not correlated to body mass index (BMI). Results suggested that even long after re-feeding has occurred, AN may be associated with moderate to severe cerebral blood flow hypoperfusion in several regions [24].

Several cerebral structures are thought to be involved in AN, such as the frontal lobes (connected with deficits in executive function and central coherence), the parietal cortex (related to body image distortions), the amygdala (related to anxiety), and the striatum (connected with obsessive–compulsive behavior). It has also been suggested that a malfunction of the insula could play a central role in orchestrating the signals regarding the external environment and internal homeostasis [25,26]. Various brain-imaging techniques have been used to investigate these brain structures and their function in order to better understand AN underlying neurobiology. Nevertheless, so far literature on structural alterations in AN has yielded conflicting results on regional brain volumes in AN compared to controls, varying from bigger to smaller, and including equal volumes [14,21,27]. Among the most studied brain regions that could be implicated in eating disorders are the insula and the orbitofrontal cortex [28]. The insula is involved in processing taste and interoceptive awareness and is thought to be a key node in the pathophysiology of eating disorders [28–30]. The orbitofrontal cortex (OFC) is important in processing specific satiety sensory signals regulating food intake. Moreover, connectivity between the OFC and the insula has been shown to be central to interoceptive and taste processing [31]. A number of structures in the insula either connect or send signals to portions of the limbic system, suggesting a key relationship between the two structures. Within this framework, a seminal study using SPECT suggested an imbalance in neural pathways or circuits, possibly within the limbic system, in early-onset AN [32].

Friederich et al. [23] showed that patients with AN, compared to healthy controls (HC), showed decreased GM volumes in the frontal lobes. This finding was confirmed by Lavagnino et al. [33,34]. Mühlau et al. [11] focused on the anterior cingulate cortex (ACC) and the supplementary motor area (SMA) [11]. These structural changes in areas that are involved in cognitive, motivational and emotional processing may underlie some aspects of the psychopathology of AN, such as impaired cognitive-behavioral flexibility, great concern about errors and striving for perfectionism [35,36]. Joos and colleagues [1] found similar changes in the ACC and in the frontal operculum. Reduced ACC volume was found in adolescents with restricting type AN (AN-r), in the early stages of the illness [16,37]. Other brain structures can be involved in AN. Nickel and colleagues [5] reported that patients with AN showed reduced regional GM volumes in the right hippocampus and the left middle and right inferior frontal gyri [5].

Despite the relevance and social impact of AN, and despite a considerable number of existing studies [4], our knowledge of the mechanisms underlying macrostructural brain changes is extremely poor. In this study, we propose a whole brain analysis approach to studying AN-r. We have compared global cerebral volumes (GM, WM, CSF, and total intracranial volume [TIV]) and regional GM volumes between adolescents with AN and HC. It was hypothesized that the AN group exhibits reduced global cerebral volumes and that specific brain regions are implicated in ongoing starvation. In particular, we hypothesized that frontal and insular volumes were relatively smaller in the AN-r group compared to HC regional volumetric measures.

## Methods

### Participants

A total of 48 participants (13–18 years old, Caucasian) were enrolled in the study. They comprised 24 girls with acute AN-r (AN-r group) and 24 age-matched healthy girls in the normal weight range (control group). All patients met the criteria for AN-r, defined by the fourth edition of the Diagnostic and Statistical Manual of Mental Disorders (DSM-IV) [38] and confirmed by the Italian version of the Kiddie-SADS-Present and Lifetime Version (K-SADS-PL) [39]. Patients were diagnosed according to the DSM-IV as they were enrolled in 2013–2014, since the Italian version of the DSM-5 [40] has only been available only since 2014. Moreover, before entering the study, AN-r subjects were interviewed with the Eating Disorder Inventory-3 (EDI-3) [41] for accurate phenotyping. To address psychopathological traits associated with the eating disorder, two other questionnaires were administered: the Child Behavior Checklist (CBCL 6–18) and the Youth Self Report (YSR 11–18) [42,43]. Patients and their parents completed all the tests during the first few days of the multiprofessional clinical assessment. Diagnosis was performed by a child and adolescent psychiatrist (S.M.).

Mean age in the AN group was 15.2 years (182 months, SD = 24 months), mean BMI was 14.5 kg/m^2^; SD = 1.7 kg/m^2^); disease duration ranged from a minimum of 4 months to a maximum of 60 months; mean disease duration was 16.1 months, (SD = 15.7 months), and delta BMI was 4.9 kg/m^2^ (SD = 2.6 kg/m^2^) (see Table 1). The 24 patients were all treated in the same clinical setting and were all right-handed.

**Table 1.** Comparison between participants with AN and HC in terms of socio-demographic and clinical variables, global cerebral volumes (mm^3^) and region of interest cerebral gray matter volumes (ml)

	AN group (*n* = 24)		HC group (*n* = 24)		*p* Value of the ANOVA test*
	Mean	SD	Mean	SD	
Age (months)	182	24	185	26	0.75
**BMI (kg/m** ^**2**^**)**	**14.5**	**1.7**	**21**	**2**	**0.001**
Delta BMI (kg/m^2^)	4.9	2.6	–	–	–
Disease duration (months)	16.1	15.7	–	–	–
**GM (ml)**	**650**	**40**	**680**	**340**	**0.02**
WM (ml)	440	30	460	30	0.14
**CSF (ml)**	**252**	**18**	**242**	**15**	**0.05**
TIV (ml)	1346	82	1377	79	0.18
**Left frontal lobe (ml)**	**91**	**7**	**96**	**6**	**0.006**
**Right frontal lobe (ml)**	**90**	**7**	**95**	**5**	**0.006**
Left parietal lobe (ml)	52	4	54	4	0.07
Right parietal lobe (ml)	51	4	53	3	0.07
Left limbic lobe (ml)	14.8	1.2	15.3	0.9	0.14
Right limbic lobe (ml)	14.3	1.3	14.7	0.9	0.16
Left occipital lobe (ml)	25.3	2.2	26.2	2.0	0.12
Right occipital lobe (ml)	27.1	2.4	28.3	2.2	0.06
Left temporal lobe (ml)	70	5	72	5	0.054
Right temporal lobe (ml)	70	5	71	4	0.1
**Left insula (ml)**	**7.6**	**0.7**	**8.1**	**0.5**	**0.016**
Right insula (ml)	7.3	0.7	7.7	0.6	0.07
Left caudate (ml)	3.8	0.3	3.7	0.3	0.11
Right caudate (ml)	3.7	0.3	3.6	0.3	0.17
Left putamen (ml)	3.2	0.3	3.1	0.3	0.7
Right putamen (ml)	3.3	0.3	3.3	0.4	0.8
Cerebellum (ml)	88	7	92	9	0.08
Brainstem (ml)	5.3	0.5	5.2	0.4	0.3

Abbreviations: AN, anorexia nervosa; BMI, body mass index; CSF, cerebrospinal fluid; GM, gray matter; HC, healthy controls; SD, standard deviation; WM, white matter

* Statistically significant differences are highlighted in bold*.*

Exclusion criteria included (a) anomalies detected in brain MRI; (b) neurological syndromes or focal neurological signs; (c) dysmorphic features suggestive of a genetic syndrome; (d) significant sensory impairment (e.g., blindness and deafness); (e) anthropometric parameters (height, head circumference) lying outside two SD from the mean of normal participants; (f) anamnesis of birth asphyxia, premature birth, head injury or epilepsy; (g) insufficient image quality for data processing and volume quantification; and (h) presence or history of schizophrenia.

Five patients with AN-r were premenarchal, whereas 19 subjects had secondary amenorrhea.

The Italian version of the K-SADS-PL [39] was administered to the AN-r participants. The assessment showed that 18 subjects fulfilled the criteria for Axis I mood disorder, 8 for anxiety disorder and in seven AN-r participants both conditions were present. Study participants were all in the same treatment setting and underwent the same treatment modality. Nine of them had just started psychopharmacological treatment with selective serotonin reuptake inhibitors and/or atypical antipsychotics and/or mood modulators, while the remaining 15 subjects were medication-naïve.

Twenty-four female adolescents without a history of neurological or psychiatric diseases, with a mean age of 15.4 years and a mean BMI of 21 kg/m^2^, served as control group. The control participants were selected from the database of clinical three-dimensional structural MRI available at our Institute. Age did not significantly differ between the AN-r and the control group (see Table 1). Due to the nature of the disorder, participants with AN-r displayed significantly lower BMI compared with healthy controls (see Table 1).

### The MRI acquisition protocol for children and adolescents

MRI data were acquired using a GE 1.5T Signa Neuro-optimized system (General Electric Medical Systems, Milwaukee, WI, USA) fitted with 40 mT/m high-speed gradients. The standard MRI protocol for children and adolescents included a whole-brain fast spoiled gradient-recalled acquisition in the steady-state T1-weighted series (FSPGR), collected in the axial plane yielding almost isotropic high-resolution brain images with voxel size of about 1 × 1 × 1 mm^3^. Written informed consent from a parent or guardian of each participant was obtained. The research protocol was approved by the Institutional Review Board of the Clinical Research Institute for Child and Adolescent Neurology and Psychiatry Fondazione Stella Maris (Prot. No. 05/2011, December 14, 2011). All participants gave written consent before the structural scans were acquired. The study was approved by the local ethics committee and conforms to standards of the Declaration of Helsinki.

### MRI data preprocessing and volume quantification

The brain MRI images of all participants were analyzed with statistical parametric mapping software (SPM8, Wellcome Department of Imaging Neuroscience, London, UK, available at http://www.fil.ion.ucl.ac.uk/spm as follows: (a) preprocessing of brain images and segmentation of GM, WM, and CSF [44]; (b) implementation of the diffeomorphic anatomical registration using the exponentiated lie algebra (DARTEL) algorithm to obtain a population-based brain template [45]; (c) affine transformation of the DARTEL template to the Montreal Neurological Institute (MNI) reference space and consequent diffeomorphic warping of the segmented brain tissues of each subject into MNI; and (d) standard smoothing with isotropic Gaussian kernel (*s* = 6 mm), including the modulation operation [46], to make the final statistics reflecting the local volume differences. Once the SPM preprocessing was completed, the smoothed, modulated, and normalized GM segments (warped into the MNI space) underwent a parcellation into regions of interest (ROIs) to allow the statistical analysis of regional GM volumes [47]. The ROIs of LONI Probabilistic Brain Atlas (http://www.loni.ucla.edu.Atlases/LPBA40) [48] were applied as masks to the segmented GM volumes of our data sample. The volumes of lobes (frontal, parietal, limbic, occipital, and temporal) and other structures (insula, caudate, putamen, cerebellum, and brainstem) were extracted for each subject.

### Statistical analysis

Group differences were evaluated for whole-brain volumes, including GM, WM, CSF, and the TIV, obtained in the brain segmentation step of the SPM-DARTEL preprocessing. The TIV was calculated as the sum of GM, WM, and CSF volumes. In addition, regional group differences in the GM volumes of the ROIs obtained according to the LONI parcellation scheme were estimated. Analysis of variance (ANOVA) was performed to identify any significant between-group difference in global tissue volumes and in specific ROIs. Correlation analyses to assess the relationship between relevant anatomical whole and regional brain volumes and the BMI and the duration of the disease were performed; for psychiatric comorbidities, psychopharmacological treatment, prepubertal phase or amenorrhea stratification analyses were performed. Scores of the YSR–DSM-oriented “Affective Problems” were used as a valid indicator of depression, and the correlation between altered volumes and YSR–DSM-oriented “Affective Problems” was studied. Correlation analyses were performed both between altered volumes (both absolute volume measures and volumes normalized to TIV) and BMI, and between altered volumes and YSR–DSM-oriented “Affective Problems”. The correlations between altered volumes and both BMI and YSR–DSM-oriented “Affective Problems” were investigated, considering the absolute volume measures and the volumes normalized to TIV.

## Results

**Figure 1.**
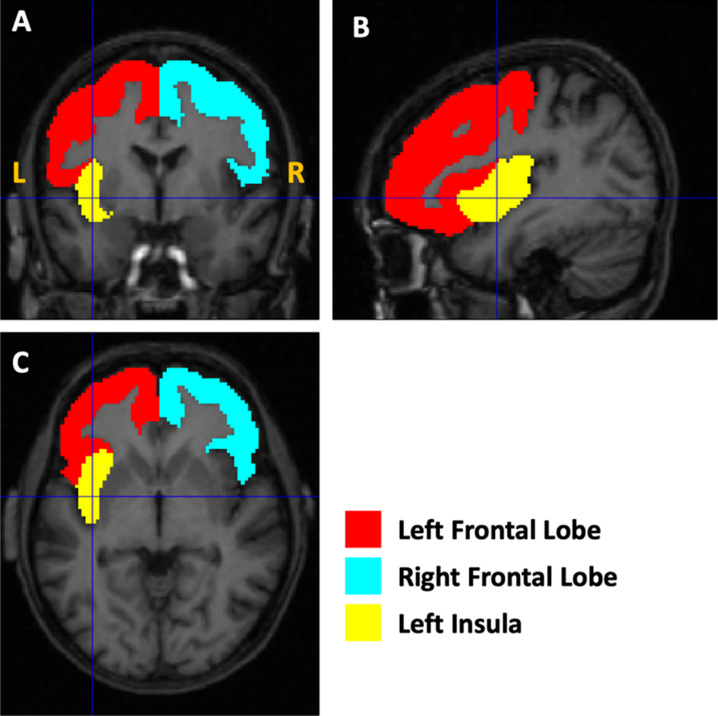
Illustration of the altered gray matter regions. The masks used to quantify the volumes of the left and right frontal lobes and the left insula are shown as an overlay on the T1-weighted magnetic resonance imaging image of the first AN subject of the cohort.

**Figure 2.**
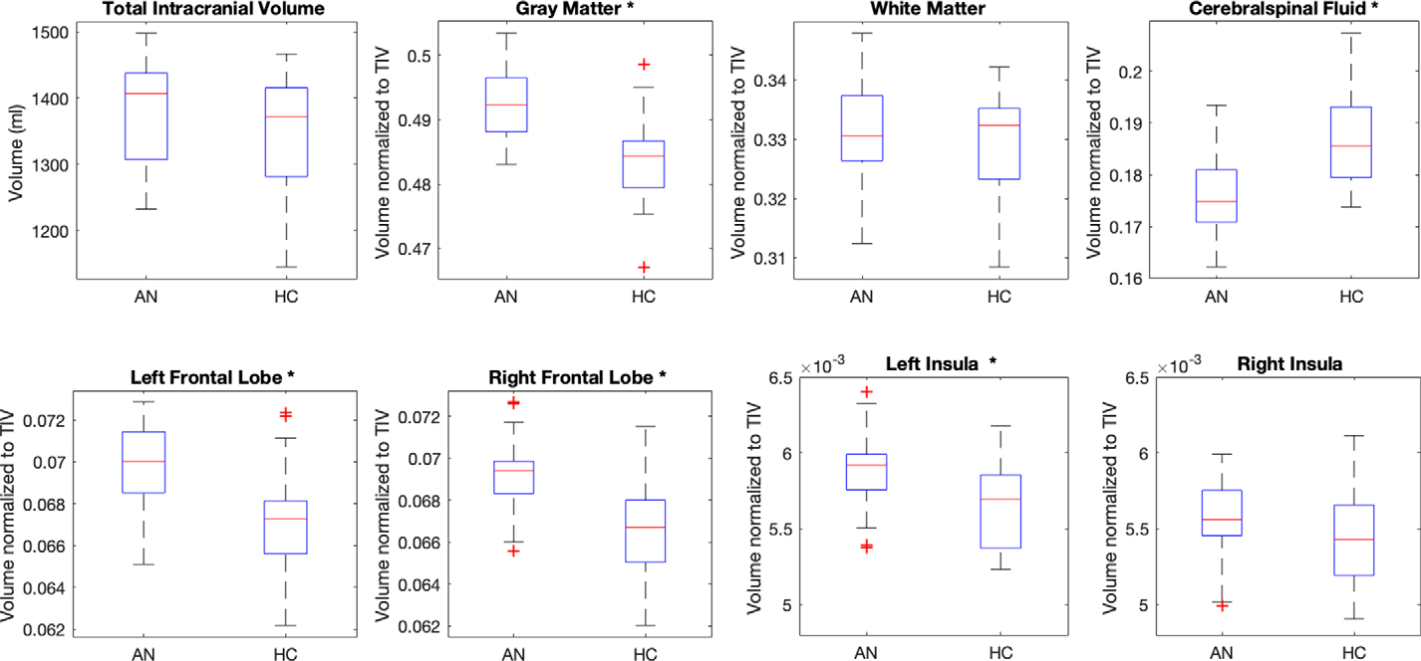
Box plots of a set of global and regional volumes obtained for the anorexia nervosa (AN) and healty controls (HC) groups of subjects. The volumes normalized to the total intracranial volume (TIV) are reported. The features showing a significant between-group difference are indicated with an “*” in the figure.

The demographic characteristics of the two groups of subjects and the results of the statistical comparison between them and all brain volumes are reported in Table 1. The morphometric analysis revealed a reduction in total GM volumes (*p* = 0.02) in participants with AN-r compared with the HC; increased CSF (*p* = 0.05) was also observed. The WM volumes did not reveal significant differences between patients with AN-r and HC. The analysis of the cerebral GM ROI volumes revealed significantly lower volumes for both frontal lobes (*p* = 0.006) and for the left insula (*p* = 0.016) in AN (see Figure 1). The box plots for a subsample of global and regional volumes obtained for the AN and the HC groups are shown in Figure 2. Neither parametric nor nonparametric correlations between relevant whole and regional brain volumes and the duration of the disease provided statistically significant results (*p* > 0.05). No relations were found between cerebral volumes and psychiatric comorbidities (*p* > 0.05), psychopharmacological treatment (*p* > 0.05), pre-pubertal phase or amenorrhea (*p* > 0.05). No significant correlations were detected between altered volumes (both absolute volume measures and volumes normalized to TIV) and BMI, or between altered volumes and YSR–DSM-oriented “Affective Problems”.

## Discussion

The aim of this study was to assess the differences in global and regional brain volumes in adolescents with AN. This study is mostly a replication of previous studies; however, it should be stressed that our findings focus on a homogenous and relatively large sample of teenage patients with AN-r.

Consistently with most studies in literature, our morphometric analysis revealed a decrease in the total GM and an increase in the CSF in patients with AN compared to HC. Conversely, no significant differences were found in the comparison of WM volumes between subjects with AN-r and controls. The finding of brain alterations involving an overall reduction of both WM and GM along with an increased volume of the ventricles and CSF is frequently reported in adult patients with AN compared to HC [5–7]. In our study, consistent with the study by Castro-Fornieles et al. [13], structural alterations were found only in the GM. This finding could be related to the early age of our sample, since higher GM plasticity in the developing adolescent brain could lead to increased susceptibility to starvation effects [4].

In our study, noteworthy volume differences were observed at a regional level. The most significant regional changes were found in right and left frontal lobes, and in the left insula. As mentioned in the introduction, previous studies have reported these regions of the brain to be the most affected in patients with AN [1,2,34,37,49,50]. Our results suggest that two main neural networks—the limbic and the frontal—are particularly relevant in AN. The insula is an integral part of the limbic circuit due to its connections with the amygdala, the anterior cingulate cortex, the orbitofrontal cortex, and the ventral striatum. A GM reduction of the insula could reflect disturbances in the limbic system, which is involved in interoceptive deficit [51], body dissatisfaction, and low self-esteem. The insular cortex represents a node of convergence for signals composing emotional and cognitive states, which emerge from the coordination between external and internal milieus, and mediating frontotemporal interaction in social and emotional context processing [25,26,52]. Moreover, the sensory aspects of taste are primarily an insula-related phenomenon, whereas higher cortical areas modulate pleasure, motivation, and cognitive aspects of taste [53,54,55]. It is worth noting that the laterality of our findings differs from the study by Zucker et al. [26], where an altered structure of the right posterior insular cortex was detected. A possible explanation for this difference could be based on the underlying imbalance in the network implicating both insular cortices, as previously suggested [56]. These authors hypothesized that a dysfunction within either insular cortex could lead to hyperactivation of the other hemisphere, which may be over- or undercompensating for the abnormal side. First, the absence of correlation between altered volumes and BMI, and the localized and unilateral differences in GM volume are unlikely to be due to the whole-brain effects of starvation.

Another functional network comprising the frontal regions that we have found to be reduced in AN is that of the executive functions: selective attention, working memory and planning, inhibition and self-control. The frontal lobes have long been associated with these functions [57]. It has been suggested that the mechanisms leading to AN are related to dysfunctions within the frontostriatal circuits, associated with the strength of the connections between frontal and subcortical areas, as well as with habit learning as in obsessive–compulsive disorder [58,59], self-regulation [60], reward processing difficulties [53], and emotion processing [61]. It has also been shown that lesions occurring in the frontal lobes can cause characteristic eating disorders. For example, Uher and Treasure [62] evaluated the relationship between lesions of various brain structures and the development of eating disorders, concluding that the latter are associated with right frontal and temporal lobe abnormalities; thus, they challenge the traditional view that eating disorders are linked to hypothalamic disturbance and suggest a major role of frontotemporal circuits in their pathogenesis [62]. From another point of view, a recent review on fMRI [63] showed a reduced activation in bottom-up pathways (e.g., in the mid-brain) in conjunction with increased top-down activation in prefrontal and orbitofrontal cortex. Additionally, this review reported aberrant activation in the insula and hypothesized that individuals with AN had an imbalance in information processing, with an impaired ability to identify the emotional significance of a stimulus (insula) and increased traffic in neural circuits concerned with planning (frontal lobes).

Third, regarding the link with starvation, our results can be interpreted via two hypotheses. According to the first hypothesis, they might indicate that the brain structures found to be damaged are more vulnerable to starvation and malnutrition than others. The second hypothesis considers the same structures to be primarily responsible for the pathophysiology of AN. Although the underlying mechanisms responsible for anatomical brain alterations in AN are not fully understood, it is often suggested that changes in the brain are attributable to malnutrition. Nevertheless, since in our study brain volume alterations were not significantly correlated with disease duration, we hypothesized that the lower regional GM volume we found is not a consequence of long abnormal nutritional status, and that it could play a role in the pathophysiology of AN.

The present results suggest that anatomical brain alteration in AN could be associated with specific traits of the illness from the very beginning. This is in agreement with earlier brain imaging studies showing that people who had recovered from AN continued to show altered activity in both the limbic and frontal networks. Although the nature of the interaction between brain functional activities and brain volumes remain unclear, the decreased whole brain volume and abnormalities in some regional brain areas might affect functional resources, causing a need for neural compensation [64]. On the other hand, our findings are in contrast with some previous studies, in which a correlation between brain atrophy and duration of illness was detected [10,65]. However, it is difficult to compare our results with these previous findings, since there are substantial differences in the age range (adolescents vs. adults) as well as in illness duration (on average 16 months vs. 7 years) of the enrolled patients [65].

Indeed, although the interplay between duration of illness and brain morphometry remains controversial, studies of adolescent samples with AN are generally less biased by illness duration.

This type of research will ultimately help predict therapeutic response and improve treatment. It is worth noting that the participants in this study were adolescents at the beginning of a severe illness and that the study was cross-sectional; thus, caution should be taken when interpreting results.

Other limitations [66] may be as follows:

- The hydration status or calories of food intake were not checked before scanning. Individuals with AN have longer periods of severe malnutrition with dehydration and food restriction and studies checking for the hydration status may be more reflective of the effects that those behaviors have on brain structure or function in this population [67,68].
- The study sample included individuals with comorbid conditions, so the effects of these conditions on the brain imaging results should be accurately assessed. However, in this research study participants were assessed using diagnostic instruments that go beyond the eating disorder diagnosis. In fact, it is important to keep in mind that AN is characterized by a broad psychopathological spectrum confirming the need to reconceptualize psychiatric comorbidity in this disorder. To address psychopathological traits associated with the eating disorder, two other questionnaires were administered, the Child Behavior Checklist (CBCL 6–18) and the Youth Self Report (YSR 11–18). The YSR provides a summary profile and a syndrome profile paralleling those of the CBCL 6–18, and CBCL and YSR scores on the Affective Problems scale corresponded closely to DSM major depressive disorder and dysthymia [69,70].
- Some patients were taking psychiatric medication. Psychoactive medications can influence brain imaging data and also need to be accounted for in the analysis [66].

Our research supports previous notions that body weight changes are associated with cerebral brain alterations, and that in adolescent patients with a short illness history, GM is more affected than WM. The volume reduction could also be a trait marker of AN, and this finding deserves to be investigated more closely with a longitudinal design study or in a study involving a remitted group. On the other hand, the results of this research showed no significant associations between the measures of brain volumes and potential confounding elements such as psychiatric comorbidities and psychopharmacological treatment and pre- or postmenarche status. The sample of adolescent patients was homogeneous from the point of view of the characteristics of the anorexic disorder, the medical conditions, and the treatment setting. This study contributes to increasing knowledge regarding the specific brain regions that could be associated with clinical features in patients with AN; our further studies will assess in follow-up evaluations whether full recovery occurs with weight restoration. Our findings support the idea that the mechanisms involved in AN are related to dysfunctions within the frontal lobes and insular cortex, to which clinical emotional and cognitive aspects of the illness, such as difficulties in reward, decision-making, and emotional and social processing, can be traced.

## Data Availability

The dataset generated and analyzed during the current study is available from the corresponding author on request.
